# Outcomes of Patients With Classic Hodgkin Lymphoma Who Relapsed After Autologous Stem Cell Transplant

**DOI:** 10.1097/HS9.0000000000000869

**Published:** 2023-04-04

**Authors:** Aung M. Tun, Yucai Wang, Aasiya Matin, David J. Inwards, Thomas M. Habermann, Ivana Micallef, Patrick B. Johnston, Luis Porrata, Jonas Paludo, Jose Villasboas Bisneto, Allison Rosenthal, Han W. Tun, James R. Cerhan, Thomas E. Witzig, Grzegorz S. Nowakowski, Stephen M. Ansell

**Affiliations:** 1 Division of Hematology, Mayo Clinic, Rochester, MN, USA; 2 Division of Hematologic Malignancies and Cellular Therapeutics, The University of Kansas, Westwood, KS, USA; 3 Internal Medicine, Division of Hematology/Oncology, Mayo Clinic Arizona, Scottsdale, AZ, USA; 4 Division of Hematology-Oncology and Blood and Marrow Transplantation and Cellular Therapy Program, Mayo Clinic, Jacksonville, FL, USA; 5 Department of Health Sciences Research, Mayo Clinic, Rochester, MN, USA

## Abstract

Immune checkpoint inhibitors (ICIs) and brentuximab vedotin (BV) are novel agents for classic Hodgkin lymphoma, including relapse after autologous stem cell transplant (ASCT). However, their impact on survival post-ASCT relapse, in comparison with conventional therapy, is less known due to the lack of randomized controlled trials. Clinical characteristics and outcomes of 115 patients with relapse (or progression) after ASCT are studied. After a median follow-up of 8.59 years from post-ASCT relapse, the median progression-free survival (PFS) and overall survival (OS) were 0.91 and 5.07 years, respectively. Median lines of therapy after post-ASCT relapse was 2 (range, 1–12). The median PFS was not reached (NR) versus 1.11 versus 0.50 versus 0.85 versus 0.78 years (*P* = 0.006) and OS was NR versus 7.60 versus 3.08 versus 3.51 versus 3.17 years (*P* = 0.28) in patients first treated with ICIs versus BV versus investigational agents versus chemotherapy versus radiation therapy (RT). First-line treatment with novel agents (ie, ICIs and BV) was associated with superior outcomes compared with investigational agents and chemotherapy/RT with a median PFS of 1.65 versus 0.50 versus 0.79 years (*P* = 0.003) and a median OS of 7.60 versus 3.08 versus 3.32 years (*P* = 0.08). Regardless of lines of therapy, the treatment with ICIs had the most favorable outcome with a median PFS and OS of 3.98 and NR years, respectively. Allogeneic stem cell transplant (allo-SCT) was done in 23 patients (20%), and the median post-allo-SCT PFS and OS were 1.31 and 2.35 years, respectively. In conclusion, survival following post-ASCT relapse improves significantly when patients receive novel agents.

## Graphical abstract

**Figure:**
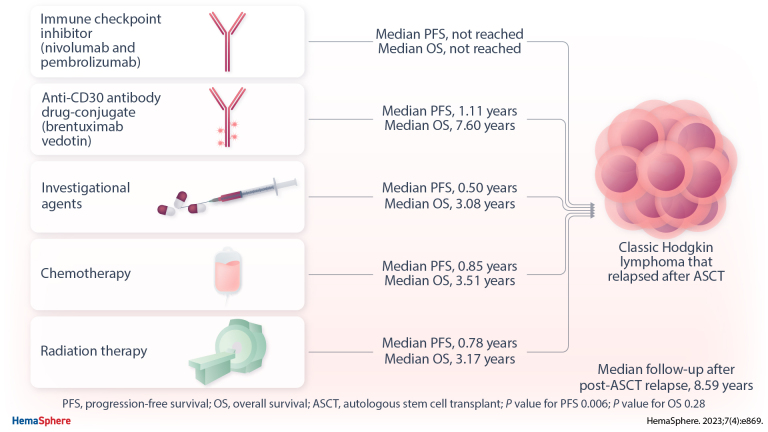

## INTRODUCTION

Classic Hodgkin lymphoma (cHL) accounts for ~10% of all lymphomas, affecting 8800 new patients annually in the United States.^1,2^ Advances in therapy have improved outcomes of cHL with ~80% of newly diagnosed patients <60 years who can expect cure with frontline therapy.^2–6^ However, while outcomes are less favorable for patients with relapsed or refractory (RR) disease, ~50% of patients with chemosensitive RR cHL can achieve long-term remission with high-dose chemotherapy/autologous stem cell transplant (ASCT).^7,8^ However, outcomes are largely unsatisfactory for patients who relapse after ASCT or those who are ineligible for ASCT.^9,10^

Traditionally, patients with post-ASCT relapse were treated with various chemotherapy regimens and radiation therapy (RT), and outcomes were generally poor.^9,11,12^ In the modern era, the landscape of RR cHL management has changed dramatically following the approval of novel therapeutic agents such as anti-CD30 antibody drug-conjugate brentuximab vedotin (BV) in 2011 and immune checkpoint inhibitors (ICIs) nivolumab in 2016 and pembrolizumab in 2017.^13–16^ Their impact, despite proven clinical benefit, has not been well established by randomized clinical trials and was not well studied in the real-world setting.^17,18^ A prior study in patients with cHL who relapsed after ASCT reported overall improvement in survival outcomes in patients treated with novel therapy. However, it was unclear whether novel agents were used as a first-line therapy or as a later line. The latter was subject to immortality time bias as a certain time period had elapsed from post-ASCT relapse to initiation of novel agents, resulting in overestimation of the survival benefit.^17,19^ Another multicenter study of patients with post-ASCT relapse, which included 4 US academic centers, reported improvement in survival outcomes in the later cohort (ie, ASCT done between 2011 and 2016) compared with the earlier cohort (ie, ASCT done between 2005 and 2010), but the impact of novel agents, after post-ASCT relapse, was not specifically addressed.^18^ Moreover, select patients may be treated with allogenic stem cell transplant (allo-SCT) after post-ASCT relapse.^20–22^ However, there is a paucity of data on the role of allo-SCT particularly in the era of novel agents.

Before the era of novel agents, clinical risk factors at post-ASCT relapse such as advanced stage disease, time to relapse, age ≥50 years, and poor performance status are associated with inferior survival outcomes.^23^ In contrast, prognostic factors are lacking in patients with post-ASCT relapse in the era of novel agents. In this study, we report clinical characteristics, treatment pattern, and outcomes of patients with cHL who relapse after ASCT from a large database over 2 decades including those treated with novel agents. We hypothesize that survival of patients with post-ASCT relapse has improved significantly with the use of novel agents (ie, ICIs and BV). We also explored the clinicopathologic characteristics that impact survival after post-ASCT relapse and clinical outcomes of patients undergoing allo-SCT.

## METHODS

### Patients

The institutional review board at Mayo Clinic approved the study. We extracted the data of consecutive adult patients who underwent ASCT for cHL at 3 Mayo Clinic sites between June 1993 and October 2017. The diagnosis of cHL was established by hematopathologists at Mayo Clinic. Clinical and pathological characteristics, as well as treatment course, clinical response to treatment (as determined by treating physician), and clinical outcome data were extracted by chart review. For this study, we included patients with RR cHL who relapsed after ASCT. Since the study was intended to evaluate treatment patterns and survival outcomes, patients who pursued hospice care or alternative medical treatment as well as those with missing medical records were excluded.

Treatment strategies after post-ASCT relapse were categorized into the following: (1) ICI; (2) BV; (3) investigational agents (excluding ICI or BV); (4) chemotherapy; and (5) RT. Detailed information of types of investigational agents and chemotherapy regimens are described in table footnotes. In this study, nivolumab, pembrolizumab, and BV are categorized as novel agents. Treatment eras (ie, post-ASCT relapse before 2011 versus 2011 and after) were determined based on patterns of subsequent treatment with novel agents and the approval of BV in patients with RR cHL by the U.S. Food and Drug Administration in 2011.

### Statistical analysis

Descriptive statistics were used to summarize and describe clinicopathologic variables. First- and second-line management strategies after post-ASCT relapse were described in the Sankey diagram. The reverse Kaplan-Meier method was used to calculate median follow-up time. Relapse (or progression), following ASCT, was defined pathologically as biopsy-proven RR cHL and clinically as per the determination of the treating physician. Progression-free survival (PFS) post-ASCT relapse was defined as the time from post-ASCT relapse until progression, relapse, or death from any cause. Overall survival (OS) post-ASCT relapse was defined as the time from post-ASCT relapse to death from any cause. PFS and OS post-ASCT relapse were plotted by Kaplan-Meier method. Two-year survival point estimates and confidence intervals (CIs) were also calculated. Cox proportional hazard models were used to evaluate associations between PFS and OS post-ASCT relapse and various clinicopathological factors at post-ASCT relapse. *P* < 0.05 was considered significant. Statistical analyses were performed using SAS software, version 15.2.1 (SAS Institute, North Carolina), and XLSTAT v2021.2. Power-user was used to draw the Sankey diagram.^24^

## RESULTS

### Clinical characteristics and treatments before post-ASCT relapse

A total of 332 patients with RR cHL who underwent salvage therapy and ASCT were identified. After a medial follow-up of 8.60 years (95% CI, 6.8-9.7), totally 136 patients had a relapse or disease progression after ASCT. Twenty-one patients were excluded due to the following reasons: missing follow-up data, n = 11; hospice care, n = 8; and alternative treatment approaches, n = 2 (Suppl. Figure S1). The remaining 115 patients with post-ASCT relapse were included in the analysis. Baseline characteristics at initial diagnosis and frontline treatment information, as well as characteristics at relapse, pre-ASCT salvage therapy and response are shown in Suppl. Tables S1 and S2. One hundred and eight (94%) patients were initially treated with doxorubicin (Adriamycin), bleomycin, vinblastine, and dacarbazine or similar chemotherapy, and RT was administered in 19 (44%) of 43 patients with limited stage disease and 13 (19%) of 67 patients with advanced stage disease. Eighteen (16%) patients had primary refractory disease (ie, disease progression during frontline therapy or persistent disease at treatment completion); 68 (59%) had relapse ≤1 year of frontline treatment completion; and 29 (25%) had relapse >1 year after the treatment completion.

Seventy-four (65%) patients received a platinum-based salvage chemotherapy regimen, and 91 (80%) had ≤1 line of salvage therapy (10 patients did not receive salvage chemotherapy based on tumor burden per treating physician: complete surgical resection, n = 1; RT, n = 1; direct transplant due to very low tumor burden, n = 8). Best response before ASCT was complete response (CR) in 28 (24%), partial response (PR) in 62 (54%), stable disease (SD) in 10 (9%), and missing in 7 (6%). Note that 8 patients (7%) who had ASCT directly without salvage therapy were not evaluable for response assessment. Best response post-ASCT was CR in 46 (40%), PR in 25 (22%), SD in 5 (4%), progressive disease in 32 (28%), and unknown/missing in 7 (6%).

### Clinical characteristics at post-ASCT relapse

The median time from day 0 of ASCT to post-ASCT relapse was 7.32 months (range, 1.56–93.72). Characteristics at post-ASCT relapse are shown in Table 1. Sixty-one patients (53%) had post-ASCT relapse before 2011 and 54 patients (47%) had post-ASCT relapse in 2011 and after. Forty-five (39%) had relapse ≤6 months from ASCT. The median age at post-ASCT relapse was 34 years (range, 20–73), and 64 patients (56%) were male. Ninety-nine (87%) had advanced stage and 59 (52%) had extranodal involvement at relapse. Fourteen (12%) had therapy with novel agents before post-ASCT relapse (salvage therapy before ASCT, n = 9; consolidative therapy after ASCT, n = 4; and used as both salvage and consolidative therapy, n = 1).

**Table 1:** Characteristics at Post-ASCT Relapse in Patients With RR cHL That Relapsed After ASCT

	N = 115	%
Time to relapse (from ASCT)		
≤6 mo	45	39
>6 mo	70	61
Year of post-ASCT relapse		
Before 2011	61	53
2011 and after	54	47
Age at post-ASCT relapse, years		
Median (range)	34 (20–73)	
≤60	110	96
>60	5	4
Sex		
Male	64	56
Female	51	44
Stage at post-ASCT relapse		
Early stage (I–II)	15	13
Advanced stage (III–IV)	99	87
Missing	1	
Extranodal sites at post-ASCT relapse		
Present	59	52
Absent	55	48
Missing	1	–
Prior therapy with ICI or BV (before post-ASCT relapse)		
Yes^a^	14	12
No	100	88

a Salvage therapy before ASCT, n = 9 (2 had both nivolumab and BV); consolidative therapy after ASCT, n = 4; and salvage and consolidative therapy, n = 1.

ASCT = autologous stem cell transplant; BV = brentuximab vedotin; cHL = classic Hodgkin Lymphoma; ICI = immune checkpoint inhibitor; RR = relapsed or refractory.

### First-line and second-line therapies for post-ASCT relapse and their outcomes

**Figure 1.**
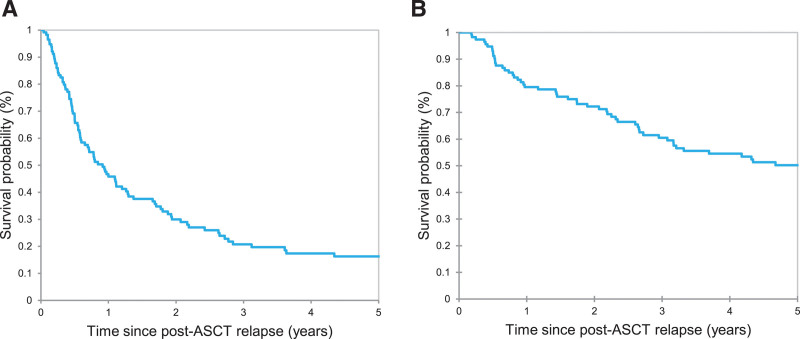
**PFS (A) and OS (B) of patients with cHL who relapsed after ASCT.** ASCT = autologous stem cell transplant; cHL = classic Hodgkin lymphoma; OS = overall survival; PFS = progression-free survival.

**Figure 2.**
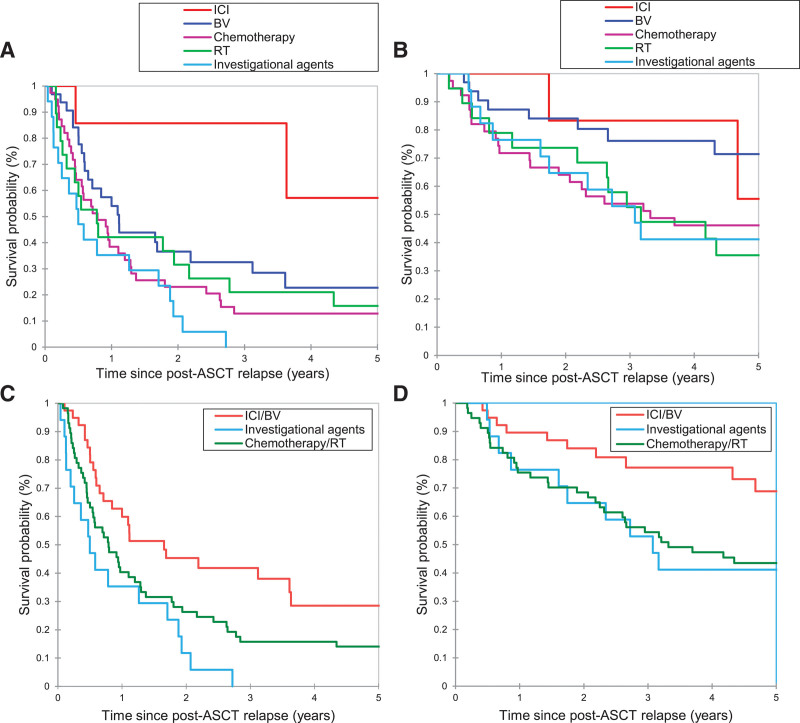
**PFS (A and C) and OS (B and D) of patients with cHL that relapsed after ASCT by the first-line treatment.** Investigational agents, everolimus, panobinostat, everolimus and panobinostat, everolimus and sorafenib, everolimus and lenalidomide, MDX-060 (iratumumab), MDX-060 plus gemcitabine, and tipifamib. ASCT = autologous stem cell transplant; BV = brentuximab vedotin; cHL = classic Hodgkin lymphoma; ICI = immune checkpoint inhibitor; OS = overall survival; PFS = progression-free survival; RT = radiation therapy.

Median follow-up after post-ASCT relapse was 8.59 years (95% CI, 6.82-9.67), and 64 (56%) deaths occurred during the follow-up period (see Suppl. Table S3 for causes of death). Median lines of therapy following post-ASCT relapse was 2 (range, 1–12). After the post-ASCT relapse, first-line therapy is summarized in Table 2. The most commonly used first-line treatment was chemotherapy (n = 38, 33%), followed by BV (n = 32, 28%), RT (n = 19, 17%), investigational agents (n = 17, 15%), and ICI (n = 8, 7%) (Table 2). Best overall response rates (ie, CR plus PR rates) were as follows: ICI, 71%; BV, 64%; investigational agents, 41%; chemotherapy, 63%; and RT, 44%. The median PFS and OS after post-ASCT relapse were 0.91 year and 5.07 years, respectively (Figure 1A and B). The median PFS after post-ASCT relapse was not reached (NR) for patients treated with ICI, 1.11 years for patients treated with BV, 0.50 year for those treated with investigational agents, 0.85 year for patients treated with chemotherapy, and 0.78 year for patients received RT (*P* = 0.006) (Figure 2A). The median OS after post-ASCT relapse was NR for patients treated with ICI, 7.60 years for BV, 3.08 years for investigational agents, 3.51 years for chemotherapy, and 3.17 years for RT (*P* = 0.28) (Figure 2B). Patients treated with ICI or BV (as a first-line therapy) had more favorable PFS and OS outcomes than patients treated with investigational agents and chemotherapy/RT (median PFS 1.65 versus 0.50 versus 0.79 years, *P* = 0.003; median OS 7.60 versus 3.08 versus 3.32, *P* = 0.08) (Figure 2C and D).

**Table 2:** First-line Treatment of 114 Patients^a^ With Post-ASCT Relapse (or Progression) and Their Outcomes

	No. of Patients (%)	Median Post-ASCT Relapse PFS in Years (95% CI)	*P* Value	Median Post-ASCT Relapse OS (95% CI)	*P* Value
ICI	8 (7)	NR (0.46-NR)	0.006	NR (1.74-NR)	0.28
BV	32 (28)	1.11 (0.60-2.19)		7.6 (4.32-NR)	
Investigational agents^b^	17 (15%)	0.50 (0.13-1.71)		3.08 (0.87-16.66)	
Chemotherapy^c^	38 (33%)	0.85 (0.46-1.2)		3.51 (1.89-7.06)	
RT	19 (17%)	0.78 (0.26-2.17)		3.17 (1.17-NR)	

a One patient who was subsequently treated with allo-SCT was not included in this analysis.

b Everolimus; panobinostat; everolimus, and panobinostat; everolimus and sorafenib; everolimus and lenalidomide; MDX-060 (iratumumab); MDX-060 plus gemcitabine; and tipifamib.

c Bendamustine; cyclophosphamide; gemcitabine; vincristine plus prednisone; ABVD; Stanford V regimen; BCVPP; ChlVPP; COPP; ESHAP; GDP; gemcitabine and oxaliplatin; gemcitabine and vinblastine; GVD; ICE; ifosfamide, gemcitabine, and vinorelbine; MOPP; ProMACE/CytaBOM.

ABVD = doxorubicin (Adriamycin), bleomycin, vinblastine, and dacarbazine; allo-SCT = allogenic stem cell transplant; ASCT = autologous stem cell transplant; BCVPP = carmustine (BCNU), cyclophosphamide, vinblastine, procarbazine, and prednisone; BV = brentuximab vedotin; ChlVPP, chlorambucil, vinblastine, procarbazine, and prednisone; CI = confidence interval; COPP = cyclophosphamide, vincristine (Oncovin), procarbazine, and prednisone; ESHAP = etoposide, methylprednisone, high-dose cytarabine, and cisplatin; GDP = gemcitabine, dexamethasone, and cisplatin; GVD = gemcitabine, vinorelbine, and liposomal doxorubicin; ICE = ifosfamide, carboplatin, and etoposide; ICI = immune checkpoint inhibitor; MOPP = nitrogen mustard, vincristine, procarbazine, and prednisone; NR = not reached; OS = overall survival; PFS = progression-free survival; ProMACE/CytaBOM = prednisone, methotrexate, Adriamycin, cyclophosphamide, etoposide, cytarabine, vincristine (Oncovin), and methotrexate; RT = radiation therapy.

**Figure 3.**
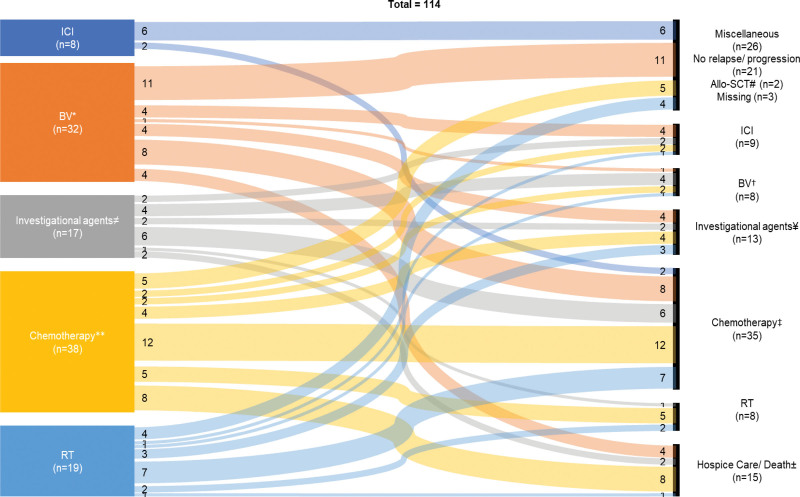
**Sankey diagram that demonstrates the first-line and second-line management of RR cHL after post-ASCT relapse.** Subsequent consolidative allo-SCT was done after: *BV (n = 4); **chemotherapy (n = 3); †BV (n = 1); ‡chemotherapy (n = 6). #2 patients had conditioning therapy and allo-SCT without preceding therapy (note 1 patient that had allo-SCT after post-ASCT relapse is not included in this diagram). ≠ and **refer to Table 2 footnote for detailed information of investigational agents and chemotherapy. ¥ and ‡ refer to Table 3 footnote for detailed information of investigational agents and chemotherapy. ± progressive disease (n = 13), pneumonitis (n = 1), allo-SCT related complication (n = 1), and unclear reason (n = 1). allo-SCT = allogeneic stem cell transplant; ASCT = autologous stem cell transplant; BV = brentuximab vedotin; cHL = classic Hodgkin lymphoma; ICI = immune checkpoint inhibitor; RR = relapsed or refractory; RT = radiation therapy.

Following the first-line treatment, disease relapse/progression occurred in 89 patients, and 76 of them had second-line therapy and 13 patients pursed hospice care. Twenty-two patients did not have a documented relapse/progression or death, whereas 4 deaths were recorded (treatment related deaths, n = 2; unknown cause of death, n = 2). The second-line management strategies in relation to first-line therapy are depicted in the Sankey diagram (Figure 3). Four patients initially treated with BV then received ICI as a second-line therapy. Allo-SCT was done in 7 patients after the first-line therapy (BV, n = 4; chemotherapy, n = 3) and 7 patients after the second-line therapy (chemotherapy, n = 6; BV, n = 1). Survival outcomes of 73 patients by the second-line treatment are summarized in Suppl. Table S4. Both PFS and OS were favorable for patients treated with ICI and RT (median PFS, NR, and 3.09 years, respectively; median OS, NR for both cohorts).

### Postrelapse survival outcomes by clinicopathological characteristics

**Figure 4.**
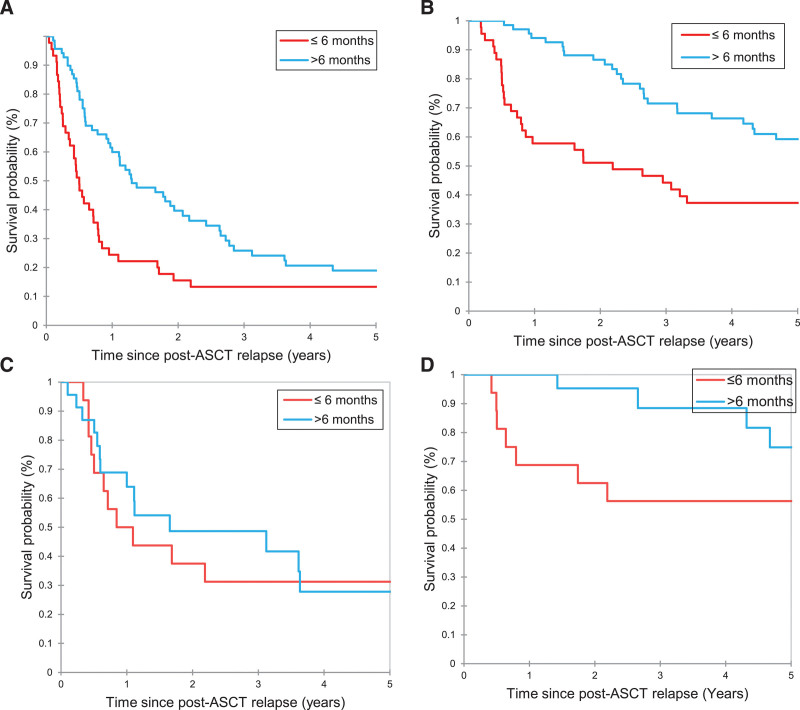
**Kaplan-Meier curves of PFS (A) and OS (B) of patients with cHL who relapsed after ASCT by time to relapse (≤6 vs >6 mo) in the entire patient population and PFS (C) and OS (D) by time to relapse (≤6 vs >6 mo) in patients treated with novel therapy following post-ASCT relapse.** ASCT = autologous stem cell transplant; cHL = classic Hodgkin lymphoma; OS = overall survival; PFS = progression-free survival.

**Figure 5.**
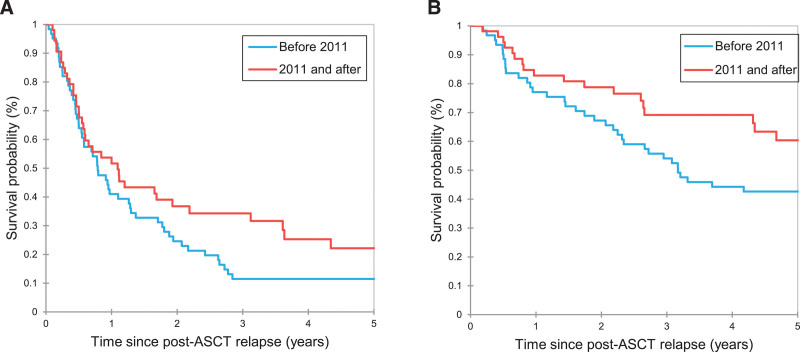
**PFS (A) and OS (B) of patients with RR cHL by era at post-ASCT relapse.** ASCT = autologous stem cell transplant; cHL = classic Hodgkin lymphoma; OS = overall survival; PFS = progression-free survival; RR = relapsed or refractory.

Survival outcomes after post-ASCT relapse by pre-ASCT clinicopathological characteristics are summarized in Suppl. Table S5. Survival outcomes after post-ASCT relapse by characteristics at post-ASCT relapse are described in Table 3. The survival outcomes were significantly better in patients who relapsed >6 months from ASCT than those who relapsed ≤6 months (median PFS 1.30 versus 0.50 years, *P* = 0.002; median OS 6.93 versus 2.19 years, *P* = 0.02) (Figure 4A and B). However, in patient treated with novel agents (ICI or BV) as a first-line therapy, PFS rates were not significantly different (median PFS 0.97 versus 1.65 years; *P* = 0.60) (Figure 4C), and there was a trend toward inferior OS in patients with a relapse within 6 months of ASCT (median OS 7.60 versus NR years; *P* = 0.06) (Figure 4D). Extranodal site involvement at post-ASCT relapse was not associated with PFS after post-ASCT relapse (median 0.72 versus 1.00 year; *P* = 0.13) but was associated with OS after post-ASCT relapse (median 3.21 versus 11.16 years; *P* = 0.01). Survival outcomes appeared better, although not statistically significant, for patients who had post-ASCT relapse in 2011 or after than those who had post-ASCT relapse before 2011 (median PFS 1.09 versus 0.79 years, *P* = 0.09; median OS 7.60 versus 3.17 years, *P* = 0.06) (Figure 5A and B).

**Table 3:** Univariate Cox Analysis of Characteristic Variables at Post-ASCT Relapse

Characteristics	Median Post-ASCT Relapse PFS in Years (95% CI)	2-Y PFS (95% CI)	HR for PFS	*P* Value	Median post-ASCT Relapse OS in Years (95% CI)	2-Y OS (95% CI)	HR for OS	*P*-Value
Time to relapse (from ASCT)								
≤6 mo (n = 45)	0.50 (0.34-0.72)	16% (5-26)	–		2.19 (0.79-7.60)	51% (37-66)	–	
>6 mo (n = 70)	1.30 (0.97-2.07)	40% (28-52)	0.53 (0.35–0.80)	0.002	6.93 (4.32-11.16)	86% (78-95)	0.54 (0.33–0.89)	0.02
Age at relapse, years								
Median (range)								
≤60 (n = 110)	0.91 (0.59-1.26)	29% (21-38)	–		5.45 (3.08-7.60)	73% (64-81)	–	
>60 (n = 5)	0.78 (0.25-4.34)	40% (0-83)	1.10 (0.44–2.71)	0.84	4.18 (0.49-NR)	60% (17-103)	1.93 (0.21–0.70)	0.25
Sex								
Male (n = 64)	0.79 (0.50-1.11)	25% (14-36)	–		4.34 (2.60-7.25)	68% (56-79)	–	
Female (n = 51)	1.12 (0.69-1.93)	36% (23-49)	0.74 (0.49–1.12)	0.16	6.93 (3.17-16.66)	78% (67-89)	0.74 (0.45–1.21)	0.23
Stage at post-ASCT relapse								
Early stage (I–II) (n = 15)	1.28 (0.26-4.34)	29 (5-53)	–		5.45 (2.25-NR)	87% (69-04)	–	
Advanced stage (III–IV) (n = 99)	0.91 (0.60-1.20)	30 (21-39)	1.17 (0.62–2.21)	0.62	4.67 (2.95-7.60)	70% (61-79)	1.14 (0.54–2.39)	0.73
Extranodal sites at post-ASCT relapse								
Present (n = 59)	0.72 (0.47-1.20)	29% (16-42)	–		3.21 (2.34-5.59)	68% (56-80)	–	
Absent (n = 55)	1.00 (0.60-1.80)	30% (18-41)	0.73 (0.47–1.14)	0.13	11.16 (4.34-NR)	78% (67-89)	0.52 (0.32–0.87)	0.01
Histology								
Nodular sclerosis (n = 85)	0.95 (0.58-1.68)	31% (21-41)	–		6.43 (3.08-7.60)	73% (63-83)	–	
Others (n = 10)	1.18 (0.42-4.34)	30% (2-58)	0.88 (0.44–1.76)		3.75 (0.64-NR)	80% (55-105)	1.22 (0.55–2.70)	
Unclassified/unknown (n = 20)	0.58 (0.25-1.20)	25% (6-44)	1.18 (0.69–2.02)	0.74	3.51 (0.81-NR)	65% (44-86)	0.97 (0.50–1.87)	0.88
Prior therapy with ICIs or BV								
Present (n = 14)	0.57 (0.29-NR)	28% (2-53)	–		2.64 (0.97-NR)	68% (41-94)	–	
Absent (n = 100)	0.94 (0.60-1.28)	31% (21-40)	0.99 (0.50–1.98)	0.99	5.45 (3.17-7.60)	73% (65-82)	0.83 (0.35–1.94)	0.67
Era at post-ASCT relapse								
Before 2011 (n = 61)	0.79 (0.54-1.26)	25 (14-35)	–		3.17 (2.25-5.59)	67% (55-79)	–	
2011 and after (n = 54)	1.09 (0.57-1.93)	37 (23-50)	0.70 (0.46–1.06)	0.09	7.60 (4.34-NR)	79% (68-90)	0.60 (0.36–1.02)	0.06

ASCT = autologous stem cell transplant; BV = brentuximab vedotin; CI = confidence interval; ICIs = immune checkpoint inhibitors; NR = not reached; OS = overall survival; PFS = progression-free survival.

In multivariate analysis adjusted for age and sex, time to relapse ≤6 months in the overall study population (hazard ratio [HR], 1.98; 95% CI, 1.30-3.02; *P* = 0.001) and first-line therapy with non-ICI/BV therapy (HR, 1.90; 95% CI, 1.19-3.04; *P* = 0.007) were associated with inferior PFS after post-ASCT relapse. Likewise, time to relapse ≤6 months (HR, 1.94; 95% CI, 1.18-3.21; *P* = 0.009), involvement of extranodal site at post-ASCT relapse (HR, 1.95; 95% CI, 1.17-3.23; *P* = 0.01), post-ASCT relapse before 2011 (HR, 1.80; 95% CI, 1.06-3.08; *P* = 0.03), and first-line therapy with non-ICI/BV therapy (HR, 2.00; 95% CI, 1.08-3.67; *P* = 0.03) were associated with worse OS after post-ASCT relapse (Table 4).

**Table 4:** Multivariate Analyses Adjusted for Age and Sex

Characteristic	HR for PFS (95% CI)	*P* Value	HR for OS (95% CI)	*P* Value
Time to relapse from ASCT ≤6 mo (vs >6 mo)	1.98 (1.30-3.02)	0.001	1.94 (1.18-3.21)	0.009
Stage at relapse advanced stage (vs early stage)	1.26 (0.66-2.41)	0.49	1.29 (0.60-2.77)	0.51
Prior therapy with ICIs or BV absent (vs present)	1.10 (0.54-2.22)	0.79	0.92 (0.38-2.19)	0.84
Extranodal sites at post-ASCT relapse present (vs absent)	1.40 (0.93-2.11)	0.11	1.95 (1.17-3.23)	0.01
First-line therapy others^a^ (vs novel agents^b^)	1.90 (1.19-3.04)	0.007	2.00 (1.08-3.67)	0.03
Era at post-ASCT relapse before 2011 (vs 2011 and after)	1.47 (0.96-2.25)	0.07	1.80 (1.06-3.08)	0.03

a Chemotherapy, RT, and investigational agents.

b Nivolumab, pembrolizumab, and BV.

ASCT = autologous stem cell transplant; BV = brentuximab vedotin; CR = complete response; PR = partial response; RT = radiation therapy; SD = stable disease.

### Outcomes by treatment modality

PFS and OS outcomes by treatment modality (ie, ICI, BV, investigational agents, chemotherapy, and RT) irrespective of prior lines of therapy were then analyzed. Lines of prior therapy and time to first initiation of respective therapy from post-ASCT relapse and median follow-up duration from initiation of respective therapy and survival outcomes are described in Suppl. Table S6 and Suppl. Figures S2–S6. The survival outcomes were most favorable for patients treated with ICI with a median PFS and OS of 3.98 and NR years, respectively.

Twenty-three (20%) patients underwent allo-SCT. Details of the clinical characteristics of the 23 patients who underwent allo-SCT are shown in Suppl. Table S7. The median time from post-ASCT relapse to allo-SCT was 1.02 years (range, 0.30–3.27) and median lines of therapy between post-ASCT relapse and allo-SCT was 1 (range, 0–7). Best response before allo-SCT was CR in 8, PR in 11, missing/not assessed in 4 patients. Six patients were treated with BV and 2 patients were treated with ICI before proceeding with conditioning therapy and allo-SCT. At a median follow-up of 5.10 years (95% CI, 2.75-7.53) after allo-SCT, 12 patients died (treatment related, n = 8; disease progression, n = 3; unknown, n = 1), while 11 patients remained alive. The median post-allo-SCT PFS and OS were 1.31 years and 2.35 years, respectively (Suppl. Figure S7).

## DISCUSSION

This is one of the largest series of patients with cHL who relapsed after ASCT.^17,18^ We found that survival outcomes of patients with post-ASCT relapse remained modest with median PFS of 0.91 year and OS of 5.07 years. However, their survival outcomes are notably more favorable than historical cohort (median OS, 1–2 y),^9,10^ likely related to variations in study populations and study era. In the first-line treatment setting, patients treated with novel agents (ICIs and BV) had far superior PFS and OS than those treated with investigational agents and chemotherapy/RT. These findings are consistent with the real-world use of novel agents, as a preferred treatment option for post-ASCT relapse. Their impact is further supported by a trend toward improvement in outcomes of patients with post-ASCT relapse in 2011 or after. These encouraging results from our study are in line with reports from pivotal clinical trials of nivolumab, pembrolizumab, and BV.^13–18^ It is, however, notable that, in the prior study of 4 US academic institutions, despite achieving a better survival outcome in the later cohort, the use of novel agents after post-ASCT relapse, as a time-dependent covariate, did not show a significant survival benefit. In a separate analysis of patients irrespective of prior lines of therapy after post-ASCT relapse, the PFS and OS of patients treated with ICIs are notably more favorable than those treated with BV, investigational agents, chemotherapy, and RT, which is in keeping with the result of the phase III study of patients with RR cHL that reported a superiority of pembrolizumab over BV in PFS.^25^

Patients treated with investigational agents, despite their fitness to be eligible, did not do better than those treated with chemotherapy/RT, emphasizing the role of disease biology and its therapeutic impact. Despite overall unfavorable prognosis of patients after post-ASCT relapse, patients in our cohort achieved a reasonable survival outcome with a median OS of ~5 years.^2^ Allo-SCT has been used as a therapy for patients with RR cHL particularly in those with post-ASCT relapse.^20–22^ However, the utility of allo-SCT is limited due to lack of consensus opinion, unclear timing, challenges in finding an appropriate donor, and concerns of all-SCT-related morbidities and mortalities.^26^ In our cohort, 20% of patients were treated with allo-SCT. The survival outcomes observed in our allo-SCT series are similar to those reported in the prior studies.^20–22^ However, due to the limited sample size, no definite conclusion can be made in regard to its role in patients after post-ASCT relapse.

Patients with a primary refractory disease or a relapse within ≤1 year of the frontline therapy, then treated with salvage therapy and ASCT, continued to have a poor outcome after post-ASCT relapse. Moreover, patients who needed ≥2 lines of salvage therapy and those who failed to achieve CR before ASCT continued to have a poor outcome after post-ASCT relapse. These suggest that there could be subsets of patients with a distinct biology that confers resistant to various therapeutic strategies. Molecular studies of tissue specimens from such patients may provide valuable insights.^27,28^ Moreover, patients who relapse ≤6 months of ASCT had worse OS, similar to the reports from the European Group for Blood and Marrow Transplantation and the Gruppo Italiano Trapianto di Midollo Osseo databases. It is, however, notable that time to post-ASCT relapse (≤6 versus >6 mo) was less relevant in patients treated with novel agents, although a larger study is needed to determine the true impact of novel agents in overcoming this high-risk disease biology. The involvement of extranodal site at post-ASCT relapse was associated with inferior OS, perhaps as a result of a higher tumor burden. Note that interpreting these prognostic variables, despite their significant associations, may be limited due to the small sample size. There is clearly a need for improvement in outcomes of such patients, and newer therapeutic strategies such as AFM13, a bispecific NK cell-engaging antibody binding CD30 on cHL cells and CD16A on NK cells and macrophages, and cellular therapies such as anti-CD30 chimeric antigen receptor T-cell therapy have been explored in patients with RR cHL.^29–31^

The strengths of our study include systematic review of consecutive cHL cases in the Mayo Clinic lymphoma database identifying detailed clinicopathological characteristics and management, a large cohort size for a relatively rare event, and a closer review of patients treated with novel agents after post-ASCT relapse. The weaknesses of the study include a retrospective study design, missing data in some patients who were lost to follow up, and a long study period (from June 1, 1993 to October 31, 2017) with different eras of management approaches, and its result should be interpreted with caution.

In summary, the outcomes of patients with cHL who relapse after ASCT have improved significantly with the use of novel agents, including those relapsing ≤6 months of ASCT. Patients treated with ICIs have the most favorable survival outcomes. Despite the advances in therapy, survival of patients with post-ASCT relapse remains modest, emphasizing the need for further advances in therapy.

## AUTHOR CONTRIBUTIONS

Conception and design: AMT, YW, and SMA; collection and assembly of data: AMT and AM; data analysis and interpretation: all authors; article writing: the first draft was prepared by AMT, YW, and SMA. All authors reviewed and revised the article; final approval of article: all authors.

## DATA AVAILABILITY

This work did not generate new data sets.

## DISCLOSURES

TH reports Data Monitoring Committee: Seagen, Tess Therapeutics, Eli Lilly & Co. Scientific Advisory Board: Morphosys, Incyte, Biegene, Loxo Oncology. Research support: Genentech, Sorrento, BMS. TH receives no compensation for these activities, institution receives compensation. Research funding (to institution): Incyte, InnoCare, LOXO Oncology, Eli Lilly, MorphoSys, Novartis, Genentech, Genmab. Advisory board (compensation to institution): Eli Lilly, LOXO Oncology, TG Therapeutics, Incyte, InnoCare, Kite, Jansen, BeiGene. Honorarium (to institution): Kite. SMA is an Associate Editor of HemaSphere. All the other authors have no conflicts of interest to disclose.

## SOURCES OF FUNDING

SMA receives research funding for clinical trials (paid to his institution) from Bristol Myers Squibb, Takeda, SeaGen, Regeneron, Pfizer, AstraZeneca, and ADC Therapeutics.

## Supplementary Material



## References

[1] Siegel RL Miller KD Fuchs HE. Cancer statistics, 2021. CA Cancer J Clin. 2021;71:7–33. 33433946 10.3322/caac.21654

[2] Ansell SM. Hodgkin lymphoma: a 2020 update on diagnosis, risk-stratification, and management. Am J Hematol. 2020;95:978–989. 32384177 10.1002/ajh.25856

[3] Connors JM Jurczak W Straus DJ. Brentuximab vedotin with chemotherapy for stage III or IV Hodgkin’s lymphoma. N Engl J Med. 2017;378:331–344. 29224502 10.1056/NEJMoa1708984 PMC5819601

[4] André MPE Girinsky T Federico M. Early positron emission tomography response-adapted treatment in stage I and II Hodgkin lymphoma: final results of the randomized EORTC/LYSA/FIL H10 trial. J Clin Oncol. 2017;35:1786–1794. 28291393 10.1200/JCO.2016.68.6394

[5] Radford J Illidge T Counsell N. Results of a trial of PET-directed therapy for early-stage Hodgkin’s lymphoma. N Engl J Med. 2015;372:1598–1607. 25901426 10.1056/NEJMoa1408648

[6] Johnson P Federico M Kirkwood A. Adapted treatment guided by interim PET-CT scan in advanced Hodgkin’s lymphoma. N Engl J Med. 2016;374:2419–2429. 27332902 10.1056/NEJMoa1510093 PMC4961236

[7] Schmitz N Pfistner B Sextro M. Aggressive conventional chemotherapy compared with high-dose chemotherapy with autologous haemopoietic stem-cell transplantation for relapsed chemosensitive Hodgkin’s disease: a randomised trial. Lancet. 2002;359:2065–2071. 12086759 10.1016/S0140-6736(02)08938-9

[8] Josting A Müller H Borchmann P. Dose intensity of chemotherapy in patients with relapsed Hodgkin’s lymphoma. J Clin Oncol. 2010;28:5074–5080. 20975066 10.1200/JCO.2010.30.5771

[9] Kewalramani T Nimer SD Zelenetz AD. Progressive disease following autologous transplantation in patients with chemosensitive relapsed or primary refractory Hodgkin’s disease or aggressive non-Hodgkin’s lymphoma. Bone Marrow Transplant. 2003;32:673–679. 13130314 10.1038/sj.bmt.1704214

[10] Arai S Fanale M DeVos S. Defining a Hodgkin lymphoma population for novel therapeutics after relapse from autologous hematopoietic cell transplant. Leuk Lymphoma. 2013;54:2531–2533. 23617324 10.3109/10428194.2013.798868

[11] Santoro A Bredenfeld H Devizzi L. Gemcitabine in the treatment of refractory Hodgkin’s disease: results of a multicenter phase II study. J Clin Oncol. 2000;18:2615–2619. 10893294 10.1200/JCO.2000.18.13.2615

[12] Moskowitz AJ Hamlin PA Jr Perales M-A. Phase II study of bendamustine in relapsed and refractory Hodgkin lymphoma. J Clin Oncol. 2013;31:456–460. 23248254 10.1200/JCO.2012.45.3308 PMC3862960

[13] Younes A Gopal AK Smith SE. Results of a pivotal phase II study of brentuximab vedotin for patients with relapsed or refractory Hodgkin’s lymphoma. J Clin Oncol. 2012;30:2183. 22454421 10.1200/JCO.2011.38.0410 PMC3646316

[14] Ansell SM Lesokhin AM Borrello I. PD-1 blockade with nivolumab in relapsed or refractory Hodgkin’s lymphoma. N Engl J Med. 2015;372:311–319. 25482239 10.1056/NEJMoa1411087 PMC4348009

[15] Younes A Santoro A Shipp M. Nivolumab for classical Hodgkin’s lymphoma after failure of both autologous stem-cell transplantation and brentuximab vedotin: a multicentre, multicohort, single-arm phase 2 trial. Lancet Oncol. 2016;17:1283–1294. 27451390 10.1016/S1470-2045(16)30167-X PMC5541855

[16] Chen R Zinzani PL Fanale MA. Phase II study of the efficacy and safety of pembrolizumab for relapsed/refractory classic Hodgkin lymphoma. J Clin Oncol. 2017;35:2125–2132. 28441111 10.1200/JCO.2016.72.1316 PMC5791843

[17] Bair SM Strelec L Nagle SJ. Outcomes of patients with relapsed/refractory Hodgkin lymphoma progressing after autologous stem cell transplant in the current era of novel therapeutics: a retrospective analysis. Am J Hematol. 2017;92:879–884. 28512788 10.1002/ajh.24792

[18] Badar T Epperla N Szabo A. Trends in postrelapse survival in classic Hodgkin lymphoma patients after experiencing therapy failure following auto-HCT. Blood Adv. 2020;4:47–54. 31899797 10.1182/bloodadvances.2019000736 PMC6960457

[19] Gleiss A Oberbauer R Heinze G. An unjustified benefit: immortal time bias in the analysis of time-dependent events. Transpl Int. 2018;31:125–130. 29024071 10.1111/tri.13081

[20] Alvarez I Sureda A Caballero MD. Nonmyeloablative stem cell transplantation is an effective therapy for refractory or relapsed hodgkin lymphoma: results of a spanish prospective cooperative protocol. Biol Blood Marrow Transplant. 2006;12:172–183. 16443515 10.1016/j.bbmt.2005.09.009

[21] Sureda A Canals C Arranz R. Allogeneic stem cell transplantation after reduced intensity conditioning in patients with relapsed or refractory Hodgkin’s lymphoma. Results of the HDR-ALLO study–a prospective clinical trial by the Grupo Español de Linfomas/Trasplante de Médula Osea (GE). Haematologica. 2012;97:310–317. 21993674 10.3324/haematol.2011.045757 PMC3269494

[22] Castagna L Bramanti S Devillier R. Haploidentical transplantation with post-infusion cyclophosphamide in advanced Hodgkin lymphoma. Bone Marrow Transplant. 2017;52:797. 28465624 10.1038/bmt.2017.26

[23] Martinez C Canals C Sarina B. Identification of prognostic factors predicting outcome in Hodgkin’s lymphoma patients relapsing after autologous stem cell transplantation. Ann Oncol. 2013;24:2430–2434. 23712545 10.1093/annonc/mdt206

[24] Power-user. Power-User Softwares. Available at: https://www.powerusersoftwares.com/advanced-charts. Accessed September 25, 2022.

[25] Kuruvilla J Ramchandren R Santoro A. Pembrolizumab versus brentuximab vedotin in relapsed or refractory classical Hodgkin lymphoma (KEYNOTE-204): an interim analysis of a multicentre, randomised, open-label, phase 3 study. Lancet Oncol. 2021;22:512–524. 33721562 10.1016/S1470-2045(21)00005-X

[26] Hoppe RT Advani RH Ai WZ. Hodgkin Lymphoma, Version 2.2020, NCCN Clinical Practice Guidelines in Oncology. J Natl Compr Canc Netw. 2020;18:755–781. 32502987 10.6004/jnccn.2020.0026

[27] Weniger MA Küppers R. Molecular biology of Hodgkin lymphoma. Leukemia. 2021;35:968–981. 33686198 10.1038/s41375-021-01204-6 PMC8024192

[28] Ansell SM. From biology to therapy: progress in Hodgkin lymphoma. Clin Lymphoma Myeloma Leuk. 2021;21:S180–S182. 10.1016/j.clml.2023.06.006 37344332

[29] Rothe A Sasse S Topp MS. A phase 1 study of the bispecific anti-CD30/CD16A antibody construct AFM13 in patients with relapsed or refractory Hodgkin lymphoma. Blood. 2015;125:4024–4031. 25887777 10.1182/blood-2014-12-614636 PMC4528081

[30] Bartlett NL Herrera AF Domingo-Domenech E. A phase 1b study of AFM13 in combination with pembrolizumab in patients with relapsed or refractory Hodgkin lymphoma. Blood. 2020;136:2401–2409. 32730586 10.1182/blood.2019004701 PMC7685206

[31] Ramos CA Grover NS Beaven AW. Anti-CD30 CAR-T cell therapy in relapsed and refractory Hodgkin lymphoma. J Clin Oncol. 2020;38:3794–3804. 32701411 10.1200/JCO.20.01342 PMC7655020

