# Supplementary material for: Outcomes of Patients With Classic Hodgkin Lymphoma Who Relapsed After Autologous Stem Cell Transplant
Source: Hemasphere. 2023 Apr 4;7(4):e869. doi: 10.1097/HS9.0000000000000869 (PMC10079336; doi:10.1097/HS9.0000000000000869)
Supplement: Supplementary file 1 [file hs9-7-e869-s001.docx]

**Supplementary Figure 1. Description of study patient selection**

Patients with RR cHL who underwent salvage therapy and ASCT (n = 332)

(N = 143)

Patients without disease relapse (n = 189)

Patients died from transplant related complications

(n = 7)

Hospice care (n = 1)

Patients who relapsed after ASCT (n = 136)

Hospice care (n = 8)

Alternative medicine (n = 2)

Treatment information missing (n = 11)

Palliative RT (n = 1)

Patients included in the analysis

(n = 115)

Abbreviation: RR, relapsed or refractory; cHL, classic Hodgkin Lymphoma; ASCT, autologous stem cell transplant.

| **Supplementary Table 1. Clinical characteristics at diagnosis and initial treatment of patients with cHL who relapsed after ASCT** | | |
| --- | --- | --- |
| Characteristics | Number of Patients (n=115) | (%) |
| **Age at initial diagnosis, years** |  |  |
| Median (range) | 29 (16–68) |  |
| ≤60 | 111 | 97 |
| >60 | 4 | 3 |
| **Sex** |  |  |
| Male | 64 | 56 |
| Female | 51 | 44 |
| **Race** |  |  |
| White | 93 | 81 |
| Others/unknown | 22 | 19 |
| **Stage at diagnosis** |  |  |
| Early stage (I-II) | 43 | 39 |
| Advanced stage (III-IV) | 67 | 61 |
| Missing/Unknown | 5 |  |
| **Bulky disease (≥7 cm)** |  |  |
| Present | 30 | 41 |
| Absent | 43 | 59 |
| Missing/Unknown | 42 | --- |
| **B symptoms at initial presentation** |  |  |
| Present | 64 | 60 |
| Absent | 43 | 40 |
| Missing | 8 | --- |
| **Sedimentation rate** |  |  |
| ≥30 mm/hour | 15 | 71 |
| <30 mm/hour | 6 | 29 |
| Missing | 94 |  |
| **International prognostic score at diagnosis** |  |  |
| 0-3 | 24 | 83 |
| ≥4 | 5 | 17 |
| Missing | 86 | --- |
| **Frontline chemotherapy** |  |  |
| ABVD (or similar) * | 108 | 94 |
| MOPP/COPP containing regimen† | 7 | 6 |
| **Limited-stage treatment** |  |  |
| Chemotherapy combined with radiation therapy (Combined modality therapy) | 19 | 44 |
| Chemotherapy alone | 24 | 56 |
| **Advanced-stage treatment** |  |  |
| Chemotherapy combined with radiation therapy | 13 | 19 |
| Chemotherapy alone | 54 | 81 |

Abbreviations: cHL, classic Hodgkin lymphoma**;** ASCT, autologous stem cell transplant; ABVD, doxorubicin (Adriamycin), bleomycin, vinblastine, and dacarbazine; MOPP, nitrogen mustard, vincristine, procarbazine, and prednisone; COPP, cyclophosphamide, oncovin, procarbazine, and prednisone.

* ABVD (or similar): ABVD; doxorubicin (Adriamycin), vinblastine, and dacarbazine (AVD) (n=1); and Stanford V.

†MOPP/COPP containing regimen: MOPP/doxorubicin (Adriamycin), bleomycin, and vinblastine (ABV) and COPP/ABV

| **Supplementary Table 2. Clinical characteristics at relapse and salvage therapy of cHL in patients included in this study (n=115)** | | |
| --- | --- | --- |
| **Time to relapse from the end of frontline therapy** |  |  |
| Primary refractory | 18 | 16 |
| ≤1 year | 68 | 59 |
| >1 year | 29 | 25 |
| **Age at ASCT, years** |  |  |
| Median (range) | 32 (19–71) |  |
| ≤60 | 111 | 97 |
| >60 | 4 | 3 |
| **Extranodal site at relapse (prior to ASCT)** |  |  |
| Present | 36 | 32 |
| Absent | 75 | 68 |
| Missing | 4 | --- |
| **Ann Arbor Stage** |  |  |
| Early stage (I-II) | 56 | 51 |
| Advanced stage (III-IV) | 54 | 49 |
| Missing | 5 | --- |
| **First line salvage regimen** |  |  |
| Platinum based regimen* | 74 | 65 |
| Gemcitabine containing regimen† | 6 | 5 |
| Novel agent containing regimen‡ | 4 | 3 |
| Others¶ | 21 | 18 |
| No salvage chemotherapy≠ | 10 | 9 |
| **Lines of salvage therapy** |  |  |
| ≤1 | 92 | 80 |
| >2 | 23 | 20 |
| **Final best response to salvage therapies (prior to ASCT)** |  |  |
| CR | 28 | 24 |
| PR | 62 | 54 |
| SD | 10 | 9 |
| NA** | 8 | 7 |
| Missing | 7 | 6 |
| **Conditioning regimen** |  |  |
| BEAM | 102 | 89 |
| CBV | 9 | 8 |
| Others | 4 | 3 |
| **Best response to conditioning therapy and ASCT** |  |  |
| CR | 46 | 40 |
| PR | 25 | 22 |
| SD | 5 | 4 |
| PD | 32 | 28 |
| Missing | 7 | 6 |
| **Radiation post-ASCT (consolidation)** |  |  |
| Yes | 26 | 23 |
| No | 87 | 77 |
| Missing | 2 | --- |
| **BV maintenance** |  |  |
| Yes | 5 | 4 |
| No | 109 | 96 |
| Missing | 1 | --- |

Abbreviations: cHL, classic Hodgkin lymphoma**;** ASCT, autologous stem cell transplant; CR, complete response; PR, partial response; SD, stable disease; PD, progressive disease; NA, not applicable; BEAM, carmustine (BCNU), etoposide, cytarabine, and melphalan; CBV, cyclophosphamide, carmustine (BCNU), and etoposide; BV, brentuximab vedotin.

*Platinum based regimen: ifosfamide, carboplatin, and etoposide (ICE); dexamethasone, high-dose cytarabine, and cisplatin (DHAP); etoposide, methylprednisone, high dose cytarabine, and cisplatin (ESHAP)

†Gemcitabine containing regimen: gemcitabine, vinorelbine, and liposomal doxorubicin (GVD)

‡Novel agent containing regimen: brentuximab vedotin; bendamustine and brentuximab vedotin; nivolumab and brentuximab vedotin

¶Others: nitrogen mustard, vincristine, procarbazine, and prednisone (MOPP); cyclophosphamide, vincristine (Oncovin), procarbazine, and prednisone (COPP); etoposide, vinblastine, and doxorubicin (EVA); cyclophosphamide, doxorubicin, vincristine, and prednisone (CHOP); etoposide, prednisone, vincristine, cyclophosphamide, and doxorubicin (EPOCH); carmustine (BCNU), cyclophosphamide, vinblastine, procarbazine, and prednisone (BCVPP); doxorubicin (Adriamycin), bleomycin, vinblastine, and dacarbazine (ABVD); prednisone, etoposide, mitoxantrone, and dacarbazine (PEND). ≠ Proceeded directly to ASCT per treating physician due to very tumor burden (n=8); proceeded to ASCT after radiation therapy (n=1); and proceeded to ASCT after surgical resection of the isolated lesion (n=1)

| **Supplementary Table 3. Causes of Death** | |  |
| --- | --- | --- |
| Causes | N=64 | % |
| Lymphoma | 49 | 77 |
| Treatment related^†^ | 11 | 17 |
| Other causes^‡^ | 1 | 2 |
| Other cancer^¶^ | 1 | 2 |
| Unknown causes | 2 | 3 |

†infection (n=5); pneumonitis (n=2); acute myeloid leukemia (n=2); graft vs host disease (n=1); and bronchiolitis obliterans (n=1)

‡congestive heart failure (n=1)

¶ metastatic melanoma (n=1)

| **Supplementary Table 4. Second line management of 73 patients* with post-ASCT relapse (or progression) and their outcomes** | | | | | |
| --- | --- | --- | --- | --- | --- |
|  | No. of patients (%) | Median post-ASCT relapse PFS in years  (95% CI) | p-value | Median post-ASCT relapse OS in years  (95% CI) | p-value |
| ICI | 9 (12) | NR (0.40-NR) | 0.005 | NR (0.4-NR) | 0.06 |
| BV | 8 (11) | 0.58 (0.16-1.60) |  | 1.91 (0.46-NR) |  |
| Investigational agents† | 13 (18) | 0.71 (0.41-2.28) |  | 6.14 (1.64-6.95) |  |
| Chemotherapy‡ | 35 (48) | 0.79 (0.35-1.33) |  | 2.44 (0.83-4.63) |  |
| RT | 8 (11) | 3.09 (0.83-NR) |  | NR (1.79-NR) |  |

*2 patients managed with allo-SCT and 1 patient with missing treatment information after relapse/progression were not included in this analysis.

Abbreviations; ASCT, autologous stem cell transplant; PFS, progression free survival; CI, confidence interval; OS, overall survival; NR, not reached; ICI, immune checkpoint inhibitor; BV, brentuximab vedotin, RT, radiation therapy.

† alpha interferon, everolimus; everolimus and panobinostat; everolimus and sorafenib; everolimus and lenalidomide; MDX-060 (iratumumab).

‡ etoposide; vinblastine; doxorubicin (Adriamycin), bleomycin, vinblastine, and dacarbazine (ABVD); cyclophosphamide, vinblastine, procarbazine, and prednisone (BCVPP); CCNU, chlorambucil, etoposide, and prednisone; dexamethasone, high-dose cytarabine, and cisplatin (DHAP); dose-adjusted etoposide, prednisone, vincristine (Oncovin), cyclophosphamide and doxorubicin (DA-EPOCH); etoposide, doxorubicin, and vinblastine; gemcitabine, dexamethasone, and cisplatin (GDP); gemcitabine, vinorelbine, and liposomal doxorubicin (GVD); gemcitabine and oxaliplatin; ifosfamide, carboplatin, and etoposide (ICE); and nitrogen mustard, vincristine, procarbazine, and prednisone (MOPP).

| **Supplementary Table 5. Survival outcomes characteristic variables at relapse before ASCT** | | | | | | | | |
| --- | --- | --- | --- | --- | --- | --- | --- | --- |
| Characteristic | Median post-ASCT relapse PFS | 2-year PFS | HR for PFS | p-value | Median post-ASCT relapse OS | 2-year OS | HR for OS | p-value |
|  | (95% CI) | (95% CI) | (95% CI) |  | (95% CI) | (95% CI) | (95% CI) |  |
| **Time to relapse** |  |  |  |  |  |  |  |  |
| Primary refractory (n=18) | 0.41 (0.23-0.95) | 17% (0-34) | ----- |  | 2.69 (0.54-NR) | 67% (45-88) | ----- |  |
| ≤1 year (n=68) | 0.78 (0.55-1.10) | 26% (16-37) | 0.71 (0.41-1.25) |  | 5.07 (2.64-10.42) | 71% (60-82) | 0.77 (0.40-1.46) |  |
| >1 year (n=29) | 1.94 (1.00-5.43) | 47% (28-65) | 0.37 (0.19-0.72) | 0.01 | 7.25 (3.17-NR) | 79% (63-94) | 0.51 (0.23-1.12) | 0.22 |
| **Age at relapse (years)** |  |  |  |  |  |  |  |  |
| ≤60 (n=111) | 0.94 (0.59-1.26) | 30% (21-39) | ----- |  | 5.45 (3.08-7.60) | 73% (65-81) | ----- |  |
| >60 (n=4) | 0.64 (0.25-3.12) | 25% (0-67) | 1.45 (0.53-3.97) | 0.46 | 2.34 (0.49-NR) | 50% (1-99) | 2.08 (0.65-6.66) | 0.22 |
| **Extranodal site at relapse** |  |  |  |  |  |  |  |  |
| Present (n=36) | 0.65 (0.46-0.78) | 26% (11-40) | ----- |  | 3.69 (1.74-10.42) | 63% 47-79) | ----- |  |
| Absent (n=75) | 1.12 (0.58-1.71) | 32% (21-42) | 0.73 (0.47-1.14) | 0.17 | 5.59 (2.95-16.66) | 76% (66-85) | 0.81 (0.48-1.36) | 0.42 |
| **Ann Arbor Stage** |  |  |  |  |  |  |  |  |
| Early state (I-II) (n=56) | 1.2 (0.54-1.80) | 31% (18-43) | ----- |  | 5.59 (2.60-16.66) | 75% (63-86) | ----- |  |
| Advanced stage (III-IV) (n=54) | 0.78 (0.55-0.95) | 29% (17-41) | 1.06 (0.70-1.60) | 0.80 | 4.18 (2.64-7.60) | 67% (55-80) | 1.13 (0.69-1.86) | 0.63 |
| **First-line salvage regimen** |  |  |  |  |  |  |  |  |
| Platinum based regimen (n=74) | 0.78 (0.55-1.11) | 28% (18-39) | ----- |  | 4.67 (2.72-NR) | 69% (58-80) | ----- |  |
| Gemcitabine containing regimen (n=6) | 1.15 (0.42-NR) | 33% (0-71) | 0.59 (0.21-1.64) |  | NR (0.64-NR) | 83% (54-113) | 0.42 (0.10-1.73) |  |
| Novel agent containing regimen (n=4) | NR (0.26-NR) | 50% (1-99) | 0.44 (0.11-1.79) |  | NR (2.60-NR) | 100% (100-100) | 0.48 (0.07-3.42) |  |
| Others (n=21) | 0.69 (0.36-1.88) | 21% (3-40) | 1.23 (0.73-2.06) |  | 2.34 (0.54-5.59) | 66% (46-87) | 1.95 (1.12-3.42) |  |
| No salvage chemotherapy (n=10) | 2.63 (0.47-NA) | 56% (23-88) | 0.57 (0.26-1.25) | 0.20 | 16.66 (1.17-NR) | 89% (68-109) | 0.42 (0.13-1.36) | 0.01 |
| **Lines of salvage therapy** |  |  |  |  |  |  |  |  |
| ≤1 (n=91) | 1.09 (0.69-1.77) | 35% (25-45) | ----- |  | 7.17 (3.69-16.66) | 74% (65-83) | ----- |  |
| ≥2 (n=23) | 0.50 (0.23-0.79) | 12% (0-27) | 1.92 (1.16-3.17) | 0.01 | 2.66 (0.52-3.32) | 65% (45-85) | 2.30 (1.32-4.10) | 0.007 |
| **Lines of salvage therapy in CR patients** |  |  |  |  |  |  |  |  |
| ≤1 (n=22) | 3.12 (0.78-NR) | 56% (35-78) | ----- |  | 6.44 (2.34-NR) | 80% (63-98) | ----- |  |
| ≥2 (n=5) | 0.78 (0.16-5.84) | 20% (0-55) | 2.11 (0.74-6.00) | 0.16 | 4.18 (0.18-NR) | 60% (17-103) | 1.93 (0.51-7.31) | 0.36 |
| **Response to salvage therapy** |  |  |  |  |  |  |  |  |
| CR (n=27) | 1.88 (0.78-4.34) | 49% (30-69) | ----- |  | 6.44 (4.18-NR) | 77% (60-93) | ----- |  |
| PR (n=61) | 0.72 (0.46-0.97) | 21% (10-31) | 2.10 (1.22-3.62) |  | 3.69 (2.60-10.42) | 68% (56-80) | 1.46 (0.74-2.86) |  |
| SD (n=10) | 0.44 (0.04-0.57) | 0% (0-0) | 5.71 (2.43-13.43) | 0.0002 | 0.97 (0.19-7.60) | 47% (14-79) | 2.98 (1.20-7.43) | 0.06 |
| **Conditioning Regimen** |  |  |  |  |  |  |  |  |
| BEAM (n=102) | 0.84 (0.58-1.30) | 32% (23-41) | ----- |  | 5.07 (3.08-11.16) | 72% (63-80) | ----- |  |
| CBV (n=9) | 1.28 (0.19-2.42) | 22% (0-49) | 1.30 (0.65-2.60) |  | 5.45 (0.19-7.06) | 78% (51-105) | 1.71 (0.81-3.62) |  |
| Others (n=4) | 0.85 (0.45-1.26) | 0% (0-0) | 1.66 (0.60-4.57) | 0.53 | 2.62 (0.54-NR) | 75% (33-117) | 1.67 (0.52-5.35) | 0.32 |
| **Radiation post-ASCT (consolidation)** |  |  |  |  |  |  |  |  |
| Yes (n=26) | 1.20 (0.46-1.94) | 29% (11-47) | ----- |  | 5.07 (2.66-16.66) | 81% (66-96) | ----- |  |
| No (n=87) | 0.80 (0.58-1.11) | 31% (21-41) | 0.88 (0.54-1.41) | 0.59 | 5.45 (3.17-10.42) | 69% (59-79) | 1.01 (0.57-1.81) | 0.96 |
| **BV consolidation post-ASCT** |  |  |  |  |  |  |  |  |
| Yes (n=5) | NR (0.46-NR) | 75% (33-117) | ----- |  | NR (1.74-NR) | 67% (13-120) | ----- |  |
| No (n=109) | 0.84 (0.58-1.20) | 29% (20-38) | 3.62 (0.50-26.05) | 0.20 | 5.07 (3.17-7.60) | 73% (64-81) | 1.19 (0.16-8.73) | 0.86 |

Abbreviations: CI, confidence interval; PFS, progression free survival; OS, overall survival; CR, complete response; PR, partial response; SD, stable disease; BEAM, carmustine (BCNU), etoposide, cytarabine, and melphalan; CBV, cyclophosphamide, carmustine (BCNU), and etoposide; ASCT, autologous stem cell transplant, BV, brentuximab vedotin.

| **Supplementary Table 6. Outcomes by treatment modality irrespective of lines of therapy after post-ASCT relapse** | | | | | | |
| --- | --- | --- | --- | --- | --- | --- |
|  | No. of patients (%) | Lines of therapy before the start of respective therapy  (range) | Median time in years from post-ASCT relapse to the start of respective therapy  (range) | Median follow up in years from respective therapy in years  (95% CI) | Median PFS in years from the start of respective therapy  (95% CI) | Median OS in years from the start of respective therapy  (95% CI) |
| ICI | 30 (26%) | 1 (0-7) | 1.40 (0.00-12.00) | 4.49 (2.39-6.24) | 3.98 (2.07-NR) | NR (4.66-NR) |
| BV | 48 (42%) | 0 (0-6) | 0.01 (range 0.00-7.13) | 6.97 (4.61-8.25) | 1.00 (0.65-1.65) | 7.60 (2.99-NR) |
| Investigational agents† | 33 (29%) | 0 (0-5) | 0.01 (range 0.00-9.60) | 9.68 (6.25-11.01) | 0.57 (0.35-1.26) | 3.40 (2.21-6.95) |
| Chemotherapy‡ | 66 (57%) | 0 (0-8) | 0.01 (range 0.00-13.62) | 8.80 (6.02-10.92) | 0.72 (0.46-1.10) | 2.90 (1.89-5.59) |
| RT | 32 (28%) | 0 (0-4) | 0.01 (range 0.00-11.52) | 10.04 (6.75-14.07) | 0.82 (0.45-1.79) | 4.34 (2.66-8.02) |

Abbreviations: ASCT, autologous stem cell transplant; ICI, immune checkpoint inhibitor; BV, brentuximab vedotin; RT, radiation therapy; CI, confidence interval; PFS, progression free survival; OS, overall survival.

† alpha interferon; everolimus; panobinostat; everolimus and panobinostat; everolimus and sorafenib; everolimus and lenalidomide; MDX-060 (iratumumab); MDX-060 plus gemcitabine; and tipifamib.

‡ bendamustine; cyclophosphamide; gemcitabine; vinblastine; vinorelbine; vincristine plus prednisone; doxorubicin (Adriamycin), bleomycin, vinblastine, and dacarbazine (ABVD); Stanford V regimen; carmustine (BCNU), cyclophosphamide, vinblastine, procarbazine, and prednisone (BCVPP); chlorambucil, vinblastine, procarbazine, and prednisone (ChlVPP); cyclophosphamide, vincristine (Oncovin), procarbazine, and prednisone (COPP); etoposide, methylprednisone, high dose cytarabine, and cisplatin (ESHAP); gemcitabine, dexamethasone, and cisplatin (GDP); gemcitabine and oxaliplatin; gemcitabine and vinblastine; gemcitabine, vinorelbine, and liposomal doxorubicin (GVD); ifosfamide, carboplatin, and etoposide (ICE); Ifosfamide, gemcitabine, and vinorelbine; nitrogen mustard, vincristine, procarbazine, and prednisone (MOPP); prednisone, methotrexate, Adriamycin, cyclophosphamide, etoposide, cytarabine, vincristine (Oncovin), and methotrexate (ProMACE/CytaBOM); dexamethasone, high dose cytarabine, and cisplatin (DHAP).

| **Supplementary Table 7. Summary of the 23 cases treated with allo-SCT** | | | | | | | | |
| --- | --- | --- | --- | --- | --- | --- | --- | --- |
| Age at post-ASCT relapse/gender | Lines of treatment for post-ASCT relapse | Conditioning regimen | Disease status prior to allo-SCT | Donor type | Progression free survival after allo-SCT  (year) | Subsequent lines of therapy (for relapse after Allo) | Overall survival after allo-SCT  (year) | Vital status  (cause of death) |
| 23/M | 1. BV | Fludarabine, BCNU, melphalan (RIC) | CR | Matched unrelated donor | NA | NA | 8.77 | Alive |
| 26/F | 1. BV | Fludarabine and melphalan | CR | Matched related donor | NA | NA | 8.59 | Alive |
| 27/F | 1. BV | Fludarabine and Busulfan | PR | Matched unrelated donor | 2.19 | NA | 2.19 | Dead (treatment related toxicities/ Bronchiolitis obliterans) |
| 31/M | 1. GDP | Fludarabine and Busulfan | PR | Mismatched related donor (8/10) | NA | NA | 5.81 | Alive |
| 32/F | 1. BV |  | PR | Matched unrelated | NA | NA | 0.34 | Alive |
| 23/M | 1.NA | Cyclophosphamide/TBI | NA | Matched related donor | 0.12 | 1. Hospice care | 0.12 | Dead  (disease progression) |
| 51/F | 1. GVD | Melphalan and fludarabine | CR | Matched related donor | 0.83 | 1. Donor lymphocyte infusion | 1.42 | Dead (treatment related toxicities/invasive Aspergilosis) |
| 28/M | 1. COPP | Cyclophosphamide/TBI | PR | Matched related donor | 0.65 | 1. Everolimus and sorafenib 2. Brentuximab vedotin 3. Panobinostat and everolimus 4. Bendamustine 5. Radiation therapy 6. ChlVPP 7. Pembrolizumab | 11.49 | Alive |
| 60/M | 1. BV 2. Nivolumab 3. Bendamustine | Fludarabine and melphalan | CR | Matched related donor | 0.54 | 1. Brentuximab vedotin 2. Revlimid | 0.90 | Alive |
| 24/M | 1. BV 2. Nivolumab 3. Bendamustine | Fludarabine and melphalan | CR | Matched related donor | NA | NA | 3.02 | Alive |
| 51/F | 1. Nivolumab 2. GVD and nivolumab 3. CAR-T 4. Liposomal doxorubicin 5. Everolimus 6. Pembrolizumab 7. ICE | Fludarabine and melphalan | PR | Matched unrelated donor | 0.15 | NA | 0.15 | Dead (treatment related toxicities/ graft vs host disease of the gut, CMV viremia and VRE bacteremia) |
| 49/M | 1. BV 2. BV plus rituximab 3. ESHAP 4. Nivolumab and ipilimumab | Fludarabine and busulfan | PR | Matched related donor | 1.31 | NA | 1.31 | Dead (treatment related toxicities/ graft vs host disease of the liver and hepatic encephalopathy) |
| 20/M | 1. BV 2. Gemcitabine 3. Radiation therapy 4. Nivolumab | Fludarabine, cyclophosphamide, and TBI | PR | Matched related donor | 1.83 | 1. Ruxolitinib and nivolumab | 2.75 | Alive |
| 39/M | 1. Radiation therapy 2. GDP 3. Nivolumab 4. Everolimus 5. Bendamustine | Fludarabine and melphalan | CR | Matched related donor | 0.54 | 1. Lenalidomide 2. Radiation therapy | 1.88 | Dead (disease progression) |
| 36/M | 1. ABVD 2. Radiation therapy 3. CHLVPP 4. DHAP 5. Vinblastine | Cladribine, thiotepa, and thymoglobulin | PR | Matched related donor | 0.42 | NA | 0.42 | Dead  (treatment related toxicities/pulmonary infection) |
| 34/M | 1. Chemotherapy plus radiation therapy (missing detailed info) |  | Missing | Missing | NA | NA | 4.32 | Alive |
| 38/F | 1. BV | Fludarabine, BCNU, melphalan | PR | Unrelated donor (9/10 match) | NA | NA | 7.53 | Alive |
| 34/M | 1. BV 2. Bendamustine plus BV | Fludarabine and TBI | CR | Haploidentical stem cell transplant | NA | NA | 1.24 | Alive |
| 48/F | 1. Radiation therapy 2. ABVD and ICE | Fludarabine and melphalan | CR | Matched related donor | 0.16 | NA | 0.16 | Dead (sudden cardiac arrest 57 days post allo-SCT) |
| 25/F | 1. ChlVPP 2. EPOCH | Missing | Missing | Missing | 4.57 | Missing | 4.57 | Dead (unknown) |
| 31/F | 1. Radiation therapy 2. Etoposide, doxoribucin, and vinblastine | Busulfan and cyclophosphamide | Not assessed | Matched related donor | 0.46 | NA | 0.46 | Dead (treatment related toxicities/pulmonary infection 167 days post allo-SCT) |
| 27/F | 1. Everolimus 2. GVD | Fludarabine and melphalan (RIC) | PR | Matched related donor | 0.50 | 1. Brentuximab vedotin 2. BCVPP 3. Everolimus and panobionstat | 1.61 | Dead (disease progression) |
| 27/M | 1. Vinblastine 2. Gemcitabine and cisplatin | Fludarabine and melphalan (RIC) | PR | Matched unrelated donor | 0.77 | 1. Vinblastine 2. Bendamustine | 2.35 | Dead (treatment related toxicities/ graft vs host disease with E. Coli bacteremia) |

Abbreviations: M, male; F, female; ASCT, autologous stem cell transplant; allo-SCT, allogeneic stem cell transplant; RIC, reduced intensity conditioning; NA, not applicable; CR, complete response; PR, partial response; SD, stable disease; BV, brentuximab vedotin; GDP, gemcitabine, dexamethasone, and cisplatin; TBI, total body irradiation; GVD, gemcitabine, vinorelbine, and liposomal doxorubicin; COPP, cyclophosphamide, oncovin, procarbazine, and prednisone; ChlVPP, chlorambucil, vinblastine, procarbazine, and prednisone; CAR-T, chimeric antigen receptor T-cell therapy; ICE, ifosfamide, carboplatin, and etoposide; ESHAP, etoposide, methylprednisone, high dose cytarabine, and cisplatin; ABVD, doxorubicin (Adriamycin), bleomycin, vinblastine, and dacarbazine; DHAP, dexamethasone, high dose cytarabine, and cisplatin; EPOCH,etoposide, prednisone, vincristine, cyclophosphamide, and doxorubicin (EPOCH); BCVPP; carmustine (BCNU), cyclophosphamide, vinblastine, procarbazine, and prednisone.

Supplementary Figure 2. Kaplan-Meier curves of PFS (A) and OS (B) of patients with cHL treated with ICIs after post-ASCT relapse

Abbreviations: PFS, progression-free survival; OS, overall survival; cHL, classic Hodgkin lymphoma; ICIs, immune checkpoint inhibitors; ASCT, autologous stem cell transplant.


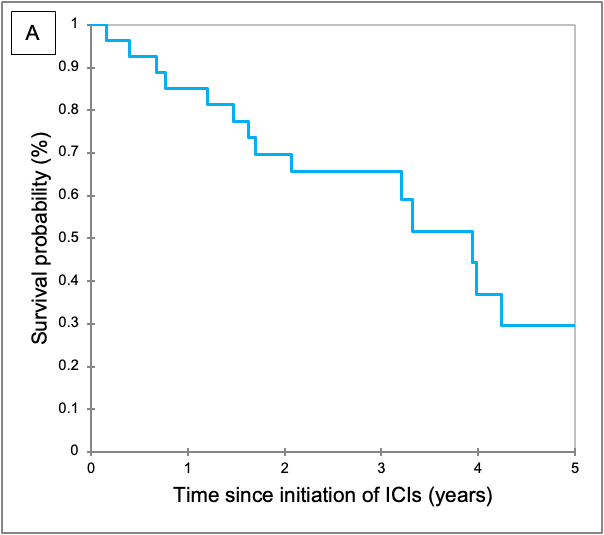

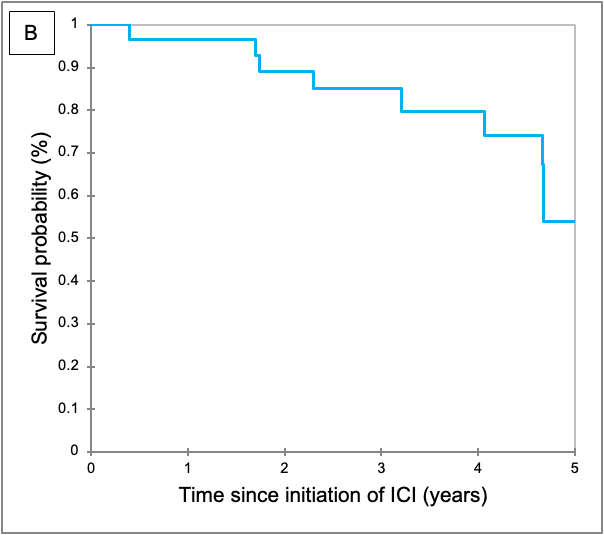


Supplementary Figure 3. Kaplan-Meier curves of PFS (A) and OS (B) of patients with cHL treated with BV after post-ASCT relapse

Abbreviations: PFS, progression-free survival; OS, overall survival; cHL, classic Hodgkin lymphoma; BV, brentuximab vedotin; ASCT, autologous stem cell transplant.


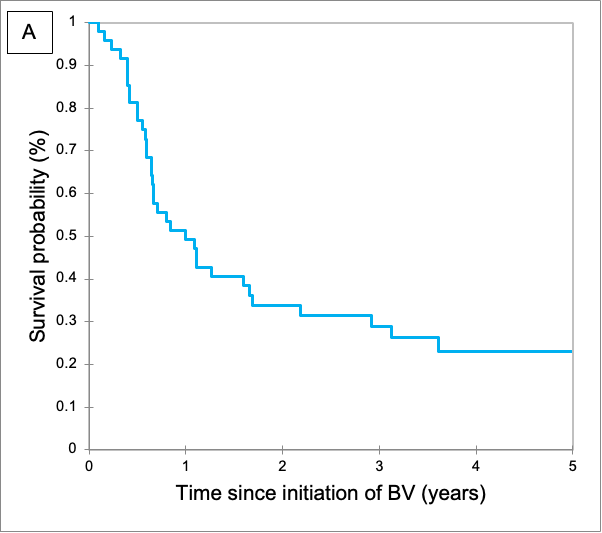

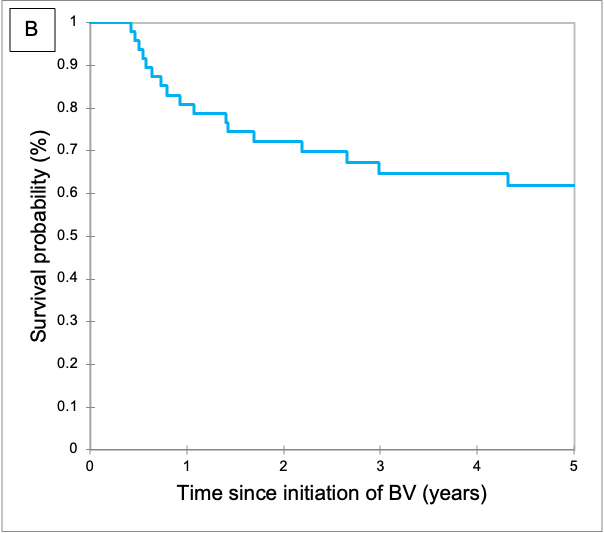


Supplementary Figure 4. Kaplan-Meier curves of PFS (A) and OS (B) of patients with cHL treated with investigational agents after post-ASCT relapse

Abbreviations: PFS, progression-free survival; OS, overall survival; cHL, classic Hodgkin lymphoma; ASCT, autologous stem cell transplant.


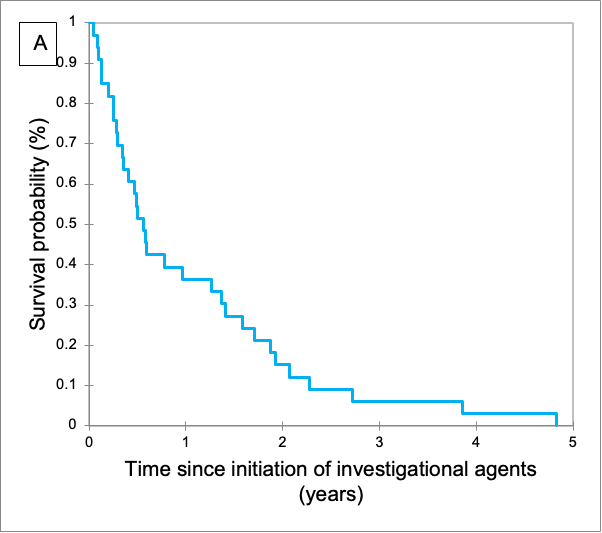

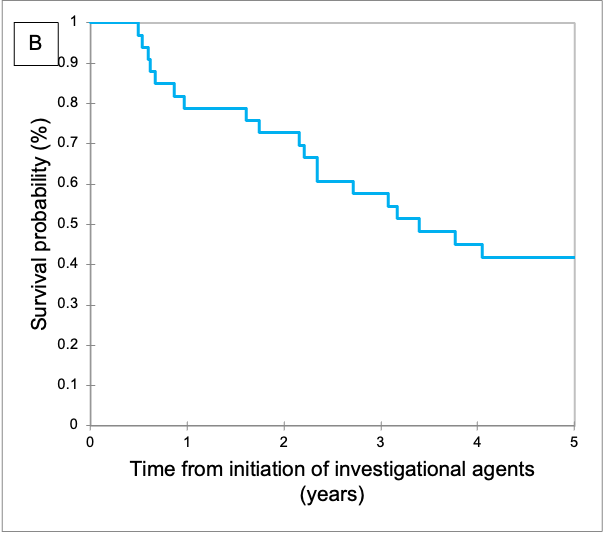


Supplementary Figure 5. Kaplan-Meier curves of PFS (A) and OS (B) of patients with cHL treated with chemotherapy after post-ASCT relapse

Abbreviations: PFS, progression-free survival; OS, overall survival; cHL, classic Hodgkin lymphoma; ASCT, autologous stem cell transplant.

Supplementary Figure 6. Kaplan-Meier curves of PFS (A) and OS (B) of patients with cHL treated with RT after post-ASCT relapse

Abbreviations: PFS, progression-free survival; OS, overall survival; cHL, classic Hodgkin lymphoma; RT, radiation therapy; ASCT, autologous stem cell transplant.

Supplementary Figure 7. Kaplan-Meier curves of PFS (A) and OS (B) of patients with cHL who had allo-SCT after post-ASCT relapse

Abbreviations: PFS, progression-free survival; OS, overall survival; cHL, classic Hodgkin lymphoma; allo-SCT, allogeneic SCT; ASCT, autologous stem cell transplant.
